# Research progress of placental vascular pathophysiological changes in pregnancy-induced hypertension and gestational diabetes mellitus

**DOI:** 10.3389/fphys.2022.954636

**Published:** 2022-07-19

**Authors:** Jiahui Lei, Meng Zhao, Lingjun Li, Bingyu Ji, Ting Xu, Miao Sun, Jie Chen, Junlan Qiu, Qinqin Gao

**Affiliations:** ^1^ Institute for Fetology, The First Affiliated Hospital of Soochow University, Suzhou, China; ^2^ Department of Obstetrics and Gynecology, The First Affiliated Hospital of Soochow University, Suzhou, China; ^3^ Department of Oncology and Hematology, The Affiliated Suzhou Science and Technology Town Hospital of Nanjing Medical University, Suzhou, China

**Keywords:** PIH, GDM, placental blood vessels, pathological changes, morphological changes

## Abstract

The placenta is a vital organ for fetal development, providing the fetus with nutrients, oxygen, and other important factors. Placenta is rich in blood vessels. Abnormal placental vascular function and blood circulation may lead to insufficient blood supply to the fetus in the uterus, leading to serious consequences such as pregnancy complications, fetal distress and even stillbirth. Pregnancy-induced hypertension (PIH) and gestational diabetes mellitus (GDM) are common complications of pregnancy. Recent studies report that pregnancy complications are often accompanied by changes in placental vascular structure and function. What are the physiological characteristics of human placental blood vessels? What are the pathological changes in the state of PIH and GDM? What are the relationships between these pathological changes and the occurrence of these pregnancy complications? Answers to these questions not only increase the understanding of placental vascular characteristics, but also provide important information for revealing the pathological mechanism of PIH and GDM. This article will summarize the research on the pathological changes of placental blood vessels in PIH and GDM, hoping to further unravel the physiological and pathological characteristics of placental blood vessels in the state of PIH and GDM, provide information for guiding clinical treatment for PIH and GDM.

## Introduction

Human placenta is a combination of embryo/fetus and maternal tissue to which the fetus is connected by an umbilical cord. Human placental villi begin to appear from 7 to 8 days after fertilization, and form a complete placenta at 3 months of pregnancy, and gradually grow with increased gestation. Placenta is a vascular organ and constitutes the active interface between the maternal and fetal blood circulation. Its normal blood supply is the key to fetal development and maternal health. The complete placental vascular bed is rich in a large number of blood vessels, which includes the chorionic plate (fetal stem villi) artery and its branches, villi and veins. Placenta is a low resistance vascular system, and plays a key role in maintaining adequate blood flow and blood volume with the placenta. Regulation of placental vascular tension is controlled by molecules brought by the blood supply, because of lack of sympathetic innervation in placental vessels (Walker and McLean, 1971). Vasoactive substances (including prostaglandin, endothelin, renin-angiotensin system, etc.) in placental tissue and surrounding blood circulation play a crucial role in controlling placental vascular function and placental blood circulation (Mak et al., 1984; Walters and Boura, 1991). As an endocrine organ, placenta can also secrete these vasoactive substances, which enter the blood circulation and play decisive roles in the stability of placental blood circulation (Gude et al., 2004; Gao et al., 2018; Myatt and Thornburg, 2018).

Insufficient blood supply to the placenta is closely related to the occurrence of pregnancy complications (Bakrania et al., 2020). Pregnancy-induced hypertension (PIH) and gestational diabetes mellitus (GDM) are common pregnancy complications. PIH is defined as systolic blood pressure (SBP) > 140 mmHg and/or diastolic blood pressure (DBP) > 90 mmHg during pregnancy (Dietl and Farthmann, 2015; Charles et al., 2017), and mainly divided into the following four types: 1) chronic hypertension, 2) gestational hypertension, 3) pre-eclampsia-eclampsia and 4) chronic hypertension with preeclampsia (Hypertension in pregnancy, 2013; NICE guideline, 2019). PIH is the main cause of morbidity and mortality in pregnant women and perinatal infants, and its incidence rate has been increasing in recent years (Mayrink et al., 2018; Qu and Khalil, 2020). Studies have shown that up to 15% of women of childbearing age suffer from PIH during pregnancy and the prevalence of preeclampsia in Europe is as high as 2.3–3% (Schneider et al., 2011; Kintiraki et al., 2015). Preeclampsia was defined as new onset of persistent hypertension (SBP/DBP≥140/90 mmHg) combined with proteinuria (≥300 mg/24-h urine) after the 20th week of pregnancy. Based on disease manifestations occurring whether before or after 34 gestational weeks, patients with preeclampsia were divided into two subgroups as early-onset and late-onset preeclampsia. In addition, late-onset and early-onset preeclampsia have different effects on the mother and fetus. For example, studies have shown that early-onset preeclampsia is more common than late-onset preeclampsia, with greater likelihood of dysfunction, placental abruption, pleural effusion, and postpartum hemorrhage (Gong et al., 2012). There are also studies showing molecular differences between early-onset and late-onset preeclampsia, and there may be no placenta-specific causative factor for late-onset mild preeclampsia (Ren et al., 2021). Although its pathogenesis is still unclear, it is widely recognized that insufficient blood supply to the placenta (placental ischemia) is the key to its pathogenesis (Spradley et al., 2019; Bakrania et al., 2020). This is because the ischemic placenta can secrete a large number of hormones and cytokines (such as soluble fms-like tyrosine kinase-1 and inflammatory factors, etc.). These factors enter the maternal blood circulation resulting in maternal vascular and renal dysfunction, and further classical PIH clinical manifestations, such as high blood pressure and proteinuria (Granger et al., 2002; Rahardjo et al., 2014).

GDM has traditionally been defined as any degree of glucose intolerance first identified during pregnancy (The Expert Committee on the Diagnosis and Classification of Diabetes Mellitus, 1997). In 2018, the American Diabetes Association (ADA) proposed a new definition of diabetes mellitus diagnosed in the second or third trimester without apparent diabetes before pregnancy (American Diabetes Association, 2018). The incidence of GDM is also increasing year by year according to epidemiological surveys (Durnwald, 2015). However, because the diagnostic criteria and screening criteria have not been unified, the incidence rates in different countries and regions are very different. Incidences ranging from 1 to 30% have been reported (Damm et al., 2016; Deputy et al., 2018; McIntyre et al., 2019). A study showed that the median incidence of GDM in all continents ranged from 6.1 to 15.2%. In regions such as the Middle East and Southeast Asia, the incidence rate can even be as high as 20%. (McIntyre et al., 2019). Insulin resistance in GDM patients has a greater impact on the metabolism of the placenta, mother, and fetus (Arshad et al., 2014). Some studies reported that the morphology and function of placental blood vessels were also changed in GDM patients (Daskalakis et al., 2008; Saha et al., 2014).

Previous reports on placental ischemia in pregnancy complications tended to focus on the hypoperfusion of the spiral artery between the uterus and placenta, ignoring the role of the placental vascular function in the blood supply of the placenta. What are the physiological characteristics of human placental blood vessels and the pathological changes in the state of PIH and GDM? In this review, we will summarize the previous studies in this area, hoping to provide important information for understanding the morphological and functional changes of placental blood vessels and the management of such pregnancy complications.

## Morphological changes in pregnancy-induced hypertension and gestational diabetes mellitus placentas

Uterine spiral arteries between the uterus and placenta are extensively remodeled during placentation to ensure sufficient delivery of maternal blood to the developing fetus. A considerable amount of research has indicated that PIH is associated with poor placentation, shallow remodeling of the uteroplacental spiral arteries (Sato, 2020; Staff et al., 2022). The spiral artery in PIH typically exhibits narrow (Leiberman, 1989; VanWijk et al., 2000) or reduced vascular branches (VanWijk et al., 2000; Hirano et al., 2002; Harsem et al., 2007; Furuya et al., 2008; Furuya et al., 2011). Although the uterine spiral artery has been the focus of much concern, some studies have also focused on placental chorionic vessels (Song, 1989; Salmani et al., 2014). For example, Deepalaxmi Salmani, et al. in a study of histological changes in gestational hypertension, found that preeclampsia and eclampsia patients had lighter placentas and smaller placentas compared with normal placentas (Salmani et al., 2014). In addition, they found that PIH placenta exhibited numerous syncytial nodules, fibrinoid necrosis, areas of calcification and hyalinization, and areas of medial hyperplasia of medium-sized blood vessels. These pathological changes may cause placental insufficiency as a result of impaired utero-placental blood flow (Salmani et al., 2014). Song L compared PIH with the normal pregnancy placenta and found that with increasing severity of PIH, the placental chorionic capillaries became more and more refined, with irregular diameters, reduced number of branches, and rough inner walls (Song, 1989). In short, vascular morphology of PIH placenta had obvious changes, and these pathological alterations may have negative implications on placental function and the growth and development of the fetus.

Regarding GDM, Soma Saha, et al. observed that the placentas of mothers with diabetes were significantly bigger in size, weight, volume, area, diameter, thickness, and circumference than those of mothers without diabetes (Saha et al., 2014). GDM placentas had significantly increased villous edema, fibrin deposition, calcification, and vascular congestion compared with controls (Saha et al., 2014). Studies by Zahra Heidari and George Daskalakis, et al. found that the placental volume, weight, intervillous space and total placental vascular volume were increased in the GDM placentas (Daskalakis et al., 2008; Heidari et al., 2019). Abdulrhmande, et al. also found that GDM placental villi had reduced vascular surface area, vascular perimeter, ratio of vascular number to villus surface area, vascular surface area to villus surface area, and vascular perimeter to villus surface area ratios compared to healthy placental villi (Dairi et al., 2020). However, there were no significant differences between diabetic and healthy villi in terms of placental barrier thickness, number of blood vessels, and villus area (Dairi et al., 2020). In addition, a study found that the villous immaturity, villous fibrinoid necrosis, and choroidal vascular hyperplasia in GDM placentas was higher than those in the normal placentas, which were closely related to placental vascular function and may be one of the important factors of fetal intrauterine hypoxia (Daskalakis et al., 2008). In conclusion, histological abnormalities were observed more frequently in the PIH and GDM placentas (Table 1).

**TABLE 1:** Morphological changes in pregnancy-induced hypertension and gestational diabetes mellitus placentas.

Morphological changes	Pregnancy-induced hypertension	Gestational diabetes mellitus
Placental appearance	Reduced volume and weight Salmani et al. (2014) (N = 50); numerous syncytial nodules, fibrinoid necrosis, areas of calcification and hyalinization Salmani et al. (2014) (N = 50)	Increase in size, weight, volume, area, diameter, thickness and perimeter Saha et al. (2014), Heidari et al. (2019) (N = 10–70)
Placental vascular bed	• Uterine spiral arteries Narrow: Leiberman, (1989); VanWijk et al. (2000) or reduced vascular branches VanWijk et al. (2000), Hirano et al. (2002); Harsem et al. (2007); Furuya et al. (2008); Furuya et al. (2011); (N = 46–49) areas of medial hyperplasia of medium-sized blood vessels Salmani et al. (2014) (N = 50)	Increased total placental vascular volume Daskalakis et al. (2008); Heidari et al. (2019) (N = 10–40)
		increased villous edema, fibrin deposition, calcification, and vascular congestion Salmani et al. (2014); (N = 70)
	• Placental chorionic capillaries: placental chorionic capillaries became more and more refined, with irregular diameters, reduced number of branches, and rough inner walls Song, (1989)	reduced vascular surface area, vascular perimeter, ratio of vascular number to villus surface area, vascular surface area to villus surface area, and vascular perimeter to villus surface area ratios Dairi et al. (2020) (N = 70);
		higher villous immaturity, villous fibrinoid necrosis, and choroidal vascular hyperplasia Daskalakis et al. (2008) (N = 40)

N, number of placentas.

## Vascular reactivity of endogenous vasoconstrictors in pregnancy-induced hypertension and gestational diabetes mellitus placenta

As we mention above, vasoactive substances (including prostanoid, histamine, serotonin, endothelin, renin-angiotensin system, etc.) in placental tissue and surrounding blood circulation play a crucial role in controlling placental vascular function and blood circulation (Mak et al., 1984; Walters and Boura, 1991). In this section, we will review the pathological alterations of the vascular reactivity to these endogenous vasoconstrictors in PIH and GDM placenta, to provide important information for understanding the abnormal placental blood supply under such pathological states (Table 2).

**TABLE 2:** Vascular reactivity in Pregnancy-induced hypertension and Gestational diabetes mellitus placental bed.

Vasoactivators	Pregnancy-induced hypertension	Gestational diabetes mellitus
prostaglandin F2	Increased sensitivity to vasoconstriction Ezimokhai et al. (1995) (N = 6)	/
	No differences between PIH and normal placenta Allen et al. (1989), Inayatulla et al. (1993) (N = 5–7)	
	Decreased sensitivity to vasoconstriction Kwek et al. (2001)(N = 5–7)	
Thromboxane A2	Decreased sensitivity to vasoconstriction Read et al. (1999); Kossenjans et al. (2000); Wareing and Baker, (2004) (N = 5–13)	Decreased sensitivity to vasoconstriction Kossenjans et al. (2000) (N = 6)
Prostacyclin	No differences between PIH and normal placenta Inayatulla et al. (1993), (Read et al. (1999) (N = 5–8)	Decreased sensitivity to vasodilation Kossenjans et al. (2000) (N = 6)
	Decreased sensitivity to vasodilation Kossenjans et al. (2000) (N = 6)	
Endothelin-1	No differences between PIH and normal placenta Inayatulla et al. (1993), (Read et al. (1999) (N = 5–8)	Decreased sensitivity to vasoconstriction Ang et al. (2001) (N = 7)
Histamine	No differences between PIH and normal placenta Szukiewicz et al. (1999) (N = 11)	/
	Decreased sensitivity to vasoconstriction Bertrand and St-Louis, (1999) (N = 9)	
Serotonin	Increased sensitivity to vasoconstriction C ruz et al. (1993), Ezimokhai et al. (1995) (N = 5–10)	Decreased sensitivity to vasoconstriction Radenkovic et al. (2009) (N = 11)
	No differences between PIH and normal placenta Allen et al. (1989), Inayatulla et al. (1993) (N = 6)	
	Decreased sensitivity to vasoconstriction Bertrand and St-Louis, (1999) (N = 9)	
Angiotensin II	Increased sensitivity to vasoconstriction Odum and Broughton Pipkin, (1989); Benoit et al. (2007) (N = 5,24)	Decreased sensitivity to vasoconstriction Kossenjans et al. (2000) (N = 6)
	No differences between PIH and normal placenta Allen et al. (1989), Inayatulla et al. (1993) (N = 6)	
	Decreased sensitivity to vasoconstriction Gao et al. (2017) (N = 55)	
Oxytocin	No differences between PIH and normal placenta Allen et al. (1989) (N = 6)	/
	Decreased sensitivity to vasoconstriction Fan et al. (2019) (N = 42)	
Vasopressin	No differences between PIH and normal placenta Ong et al. (2002) (N = 17)	/
	Decreased sensitivity to vasoconstriction Wareing and Baker, (2004), Gao et al. (2019) (N = 20)	

N, number of placentas.

### Prostanoids

Prostanoids are composed of prostaglandin (PG), thromboxane (TX) and prostacyclin (PGI2), and play an important role in the control of the umbilical-placental circulation (Tuvemo, 1980). PG and TX have vasoconstriction effects, while PGI2 has a powerful vasodilator effect (Maigaard et al., 1986; Es kildsen et al., 2014). There are two different subtypes of PG (PGE2 and PGF2). In human placental blood vessels, PGE2 has only a weak vascular response, while PGF2 can cause moderate vasoconstriction (Allen et al., 1989). During pregnancy, placental trophoblasts can produce TXA2. TXA2 is extremely unstable and can be hydrolyzed into TXB2. TXB2 is a stable metabolite of TXA2, which gradually increases with increasing gestational age (Noort et al., 1987). PGI2 is produced by arachidonic acid in vascular endothelial cells under the action of cyclooxygenase. The content of PGI2 increases as pregnancy progresses and reaches a peak in the late pregnancy (Goodman et al., 1982).

In studies of PIH and normal pregnancies, Robinson, et al. found that the content of PG in the PIH placenta was significantly lower than that in the normal placenta (Robinson et al., 1979), while Hillier and Smith, et al. reported that there was no significant difference in the content of PG between the normal and PIH placenta (Hillier and Smith, 1981). Ezimokhai, et al. found that the chorionic plate artery in PIH placenta has an increased contraction sensitivity to PGF2 (Ezimokhai et al., 1995), while Allen and Inayatulla, et al. found that there is no difference in the contraction response to PGF2 between PIH and normal placental arteries (Allen et al., 1989; Inayatulla et al., 1993). Kwek, et al. reported the maximum vasoconstrictor effect induced by PGF2 in placental resistance arteries was significantly reduced in vessels obtained from severely preeclamptic compared with normal pregnancies (Kwek et al., 2001). There was no significant difference in plasma TXB2 levels between prenatal and postpartum mothers in normal pregnancy, while the plasma TXB2 levels of postpartum mothers in the PIH group were significantly lower than that before delivery (Malatyalioglu et al., 2000). TXA2 and TXB2 in the PIH placenta were significantly higher than those in the normal placenta (Fitzgerald et al., 1990; Wang et al., 1992). Studies have shown that, compared with normal placenta, U46619 (TXA2 analog)-mediated placental vasoconstriction was significantly reduced in the PIH placenta (Kossenjans et al., 2000; Read et al., 1999). Waring et al. reported that maximal constriction of placental small arteries to U46619 was reduced in PIH (Wareing and Baker, 2004).

Compared with normal pregnancy, the content and activity of PGI2 in the PIH placental vascular tissue was significantly reduced (Remuzzi et al., 1980); while Ylikorkala et al. reported that there was no difference in the PGI2 level in both the maternal circulation and placental tissue between PIH and normal groups (Ylikorkala et al., 1981). PGI2 can cause moderate dilation in human placental blood vessels and placental lobules (Mak et al., 1984; Maigaard et al., 1986). Kossenjans, et al. found that PGI2-mediated diastolic function was decreased in the PIH placenta (Kossenjans et al., 2000). Two other studies reported that whether it is placental blood vessels or perfused placental lobules, PGI2 has no difference in the vasodilation of normal and PIH placenta (Inayatulla et al., 1993; Read et al., 1999).

In a study of GDM and normal pregnancy, Saldeen et al. found no significant difference in umbilical vascular PGI2 or TxA2 production between the GDM and the control group (Saldeen et al., 1996). However, the ratio of PGI to TXA in the veins of the diabetic/impaired glucose tolerance group was significantly lower (Saldeen et al., 1996). Umbilical artery function index was positively correlated with the ratio of PGI/TXA2 in the umbilical cord vessel segment and umbilical cord plasma TxA2 concentration. Umbilical cord plasma TxA2 concentrations were significantly elevated in patients with high umbilical cord arterial pulsatility index (Saldeen et al., 1996). This suggests that umbilical cord vascular dysfunction in diabetes/impaired glucose tolerance is associated with the PGI/TXA2 ratio, but the specific effects on vasoconstriction or relaxation are still lacking. A study by R Figueroa, et al. showed that hypoxia caused a larger PGI-independent relaxation in placental arteries and veins of women with gestational diabetes than in normal vessels (Figueroa et al., 1993). Kossenjans, et al. found that fetal-placental arteries from GDM placenta exhibited a blunted contractile response to U46619, and an attenuated vasodilatory response to PGI (Kossenjans et al., 2000).

### Endothelin-1

Endothelin-1 (ET-1) produced in endothelial cells is the most effective and long-lasting vasoconstrictor. In vascular tissue, ET-1 mediates vasoconstriction through type A (ETA) and type B receptors (ETB) (Paradis and Zhang, 2013). ET-1 is widely distributed in human placental tissue and plays an important role in regulating placental vascular tension and uteroplacental circulation (Paradis and Zhang, 2013). In normal pregnancy, plasma ET-1 decreases with the increasing gestational age, and remains at a low level until delivery (Lygnos et al., 2006). A number of studies have shown that the plasma ET-1 level of pregnant women with PIH is significantly higher than that of normal pregnant women, and the plasma ET-1 content is positively correlated with the severity of PIH (Zhao et al., 2012; Shah and Khalil, 2015). The ET-1/ETR system is altered in PIH and GDM placental tissue. Expression of ET-1, ETA and ETB was upregulated in early onset preeclamptic placentas; ET-1/ETR were down-regulated in GDM placentas (Dieber-Rotheneder et al., 2012). In the PIH placenta tissue, ET-1 level is also higher and positively correlated with the severity of symptoms (Singh et al., 2001). However, it has also been reported that the content of ET-1 in the PIH placenta is similar to that of normal pregnancy (Bernardi et al., 2015).

Currently, the results of research on the reactivity of placental blood vessels to ET-1 are limited. Only two studies reported that there is no significant difference in ET-1-mediated placental perfusion and vasoconstriction between normal pregnancy and PIH placenta (Inayatulla et al., 1993; Read et al., 1999). Serum ET-1 level in GDM was significantly higher compared to controls (Al-Ofi et al., 2021), while both Swiderski and Beata Telejko, et al. showed that plasma ET-1 levels in women with GDM were not significantly different from those observed in pregnant women without GDM (Telejko et al., 2009; Swiderski et al., 2010). Although no relevant studies have been found to prove abnormal plasma ET-1 levels in pregnant women with GDM, some studies have shown that women with GDM are less sensitive to ET-1 than pregnant women without GDM (Ang et al., 2001). Decreased sensitivity observed in pregnant women with GDM may reflect abnormal vascular reactivity (Ang et al., 2001). In limited studies, it can be speculated that the altered vascular function of pregnant women with GDM may be associated with abnormal changes in the sensitivity of plasma ET-1. However, there are few studies on the correlation between placental vasoconstriction changes and ET-1 in pregnant women with GDM, and more research is needed to draw the correlation between them.

### Histamine

Histamine (HA) has a clear role in non-placental blood vessels, which differs depending on the type of blood vessel. Generally, HA can mediate the vasoconstriction of the mesenteric and cerebral arteries (Schjerning et al., 2013; Nguyen et al., 2016), while in the retina and carotid arteries, HA exhibits a vasodilation effect (Otani et al., 2016; Yan et al., 2017). HA has been assumed to contribute to embryo-uterine interactions due to its vasoactive, differentiation and growth-promoting properties. Placenta can secrete a large amount of HA and HA-degrading enzyme Diamine oxidase (DAO) (Maintz et al., 2008). High levels of DAO can prevent excessive HA from entering the maternal or fetal circulation from the placenta. During normal pregnancy, plasma HA levels decrease with increasing gestational age, and drop to the lowest point in the second trimester (Dubois et al., 1977).

The balance between HA and DAO seems to be crucial for an uncomplicated pregnancy (Maintz et al., 2008). HA has been implicated in regulatory processes of pregnancy, and in the pathogenesis of PIH (Purcell, 1992). Two studies reported that compared with the control group, the HA concentration in the PIH placental tissue is higher, and the DAO activity is lower, which promotes the increase of HA in the maternal circulation (Maintz et al., 2008; Szewczyk et al., 2012), suggesting that HA may be related to the occurrence of PIH. Reduced DAO activities have been also found in GDM placenta (Maintz et al., 2008). Some studies indicated HA can induce concentration-dependent contractions in isolated human placental vessels (Reviriego et al., 1990; Szukiewicz et al., 1999), whereas the study by Mills, et al. reported HA can induce endothelial-dependent relaxation in human chorionic plate artery rings (Mills et al., 2007). HA can directly cause vasoconstriction by binding to HA-1 receptors, and vasodilation by directly activating HA-2 receptors on the vascular smooth muscle layer (Mills et al., 2007). Szukiewicz, et al. found that there were no differences in the increase of perfusion pressure following administration of HA between normal and PIH placenta (Szukiewicz et al., 1999). At present, there are few studies on the vascular sensitivity of placental blood vessels to HA in PIH or GDM, and only one study shows that a decreased maximal response to HA in placental vein rings from PIH with respect to those from normal pregnancies (Bertrand and St-Louis, 1999).

### Serotonin

Serotonin, also known as 5-hydroxytryptamine (5-HT), plays a crucial part in placentogenesis and fetal development (St-Pierre et al., 2016). As a strong vasoconstrictor, serotonin can also induce contraction in placental arteries and increase the perfusion pressure through 5-HT receptors (Hull et al., 1994). Balkovetz, et al. reported that there is an efficient system in the placenta to remove serotonin from the maternal circulation, which will lead to a decrease in uterine and placental vascular tone, thereby ensuring optimal blood flow to the utero-placenta (Balkovetz et al., 1989). The plasma 5-HT levels of pregnant women with PIH are significantly higher than those of women without PIH (Middelkoop et al., 1993; Carrasco et al., 1998). 5-HT in placenta tissue can be degraded by monoamine oxidase A (MAO-A). Compared with normal pregnancy, the expression and activity of MAO-A in PIH placenta are very low (Bottalico et al., 2004), suggesting that 5-HT may be closely related to PIH. Recent studies revealed that serotonin, serotonin transporter (SERT) and 5-HT (2A) receptor might play a role in the aetiology of GDM (Viau et al., 2009). Significantly decreased expressions of SERT, and 5-HT (2A) receptor were observed in placental tissues from GDM compared with normal pregnancies (Viau et al., 2009). SERT can regulates the level of 5-HT in placenta. Li, et al. found that the free plasma 5-HT levels were elevated in GDM, whereas, the 5-HT uptake rates of GDM trophoblast were significantly down-regulated, due to impairment in the translocation of SERT molecules to the cell surface (Li et al., 2014).

Currently, there are controversies about the results of studies on the response of PIH placental blood vessels to 5-HT. For example, Cruz and Ezimokhai, et al. showed that compared with the normal placenta, the sensitivity of the PIH placental chorionic plate artery and venous rings to 5-HT is significantly enhanced (C ruz et al., 1993; Ezimokhai et al., 1995). Other studies have shown that there is no significant difference in 5-HT-mediated placental vasoconstriction response between normal and PIH placenta (Allen et al., 1989; Inayatulla et al., 1993). However, studies by Bertrand, et al. have shown that the placental blood vessels in PIH have a reduced contraction response to 5-HT (Bertrand and St-Louis, 1999). In general, in the limited research on the function of placental blood vessels, there are conflicting conclusions about the response of PIH placental blood vessels to 5-HT. Studies have shown that in normal pregnancy, 5-HT produces a concentration- and endothelium-dependent constriction of human umbilical arteries, of which the greatest vascular response may be controlled by endoprosta cyclin. Compared with normal pregnancy, 5-HT has a reduced contractile effect in the umbilical arteries of pregnant women with GDM and is associated with endothelial dysfunction (Radenkovic et al., 2009). Similarly, the 5-HT uptake rates of GDM-trophoblast and the SERT expression on their surface were severalfold lower compared with control subjects (Li et al., 2016).

### Angiotensin II

The Renin-Angiotensin-System (RAS) plays an important role in the pathogenesis of hypertension (Crowley et al., 2006; Ward et al., 2022). Human placental RAS contains renin, angiotensinogen (AGT), angiotensin converting enzyme (ACE), angiotensin converting enzyme 2 (ACE2), angiotensin I (Ang I), angiotensin (1-7)) and angiotensin II (Ang II) and its type 1 receptor (AT1R) and type 2 receptor (AT2R) (Ihara et al., 1987; Valdes et al., 2006; Gao et al., 2017). ACE can convert Ang I into active Ang II, and ACE2 can promote the degradation of Ang II. Ang II is the main active substance of RAS and has the effect of constricting blood vessels via binding AT1R and AT2R (Crowley et al., 2006). During normal pregnancy, the placental RAS is activated, with an increase in AGT, renin and ACE levels, which could ultimately lead to a local increase in Ang II (Ito et al., 2002). Therefore, placental Ang II is very important for regulating placental vascular function and blood circulation.

Extensive studies have been done to determine the circulating and placental Ang II in normal and PIH pregnancies. Some studies indicated that the level of Ang II was increased, whereas others showed it unchanged or decreased in PIH (Hanssens et al., 1991; Granger et al., 2001; Gao et al., 2017). In the placenta, some published studies found that Ang II and AT1R levels were increased in PIH (Leung et al., 2001; Judson et al., 2006; Anton et al., 2008). The other reported no differences in Ang II between the normal and PIH placenta (Kalenga et al., 1996). In placental chorionic arteries, Ang II produced potent contractions. In reviewing previous studies on PIH placental vessels, placental vascular reactivities in response to Ang II are controversial. Notably, two studies showed that there were no qualitative differences in response to Ang II in chorionic plate arteries between the normal and PIH placenta (Allen et al., 1989; Inayatulla et al., 1993). Some other studies reported that the isolated human chorionic plate arteries from PIH in response to Ang II were significantly sensitive (Odum and Broughton Pipkin, 1989; Benoit et al., 2007). The reason for this controversy is that the sample size in those human studies was too small (often only 12 or even fewer). We recently used a relatively larger sample size (>50 or even 100 per group), and found that the responses of placental vessels to Ang II were significantly reduced in the PIH placenta (Gao et al., 2017).

It has been shown that the circulating RAS is activated during normal pregnancy, but little is known about RAS in pregnancies complicated by GDM. Nogueira, et al. reported that levels of Ang I, Ang II and Ang-(1-7) were higher in pregnant women, but showed a different pattern in the GDM group, in which reduced Ang-(1-7) circulating levels were found (Nogueira et al., 2007). This observation suggested that reduced levels of the vasodilator Ang-(1-7) could be implicated in the endothelial dysfunction seen in GDM women (Nogueira et al., 2007). Sugulle, et al. showed that soluble (pro)renin receptor in GDM maternal plasma is dysregulated (Sugulle et al., 2017). Zhang, et al., determined the cord blood Ang II concentration at birth, and found that the cord Ang II concentration was increased in GDM group (Zhang et al., 2013), but its effect on vasoconstriction and relaxation in GDM has not been studied. Diabetic conditions increased the vascular reactivity to Ang II in several studies (Velazquez-Roman et al., 2011). The studies about GDM placental vascular reactivity are very few. Only one study reported that the vascular response to Ang II was significantly attenuated in GDM placentas compared with controls (Kossenjans et al., 2000).

### Oxytocin and vasopressin

Oxytocin is widely used clinically. Oxytocin and its receptors are abundant in human placental tissues (Fan et al., 2019). As a vasoactive substance, oxytocin has a constrictive effect on placental blood vessels (Fan et al., 2019), suggesting that oxytocin has an important role in regulating placental vascular tension and blood circulation. However, research on the reactivity of oxytocin in the placental blood vessels of PIH or GDM is limited. A study 30 years ago reported that there is no difference between oxytocin-induced placental vasoconstriction in PIH and normal pregnancy (Allen et al., 1989). Our recent study found that compared with normal placenta, the blood vessels of the placenta in PIH show a lower contraction sensitivity to oxytocin (Fan et al., 2019). Similar to oxytocin, vasopressin is a stress hormone that maintains hemodynamic stability. In 2014, Yeung and Santillan, et al. reported that compared with normal pregnancy, the content of vasopressin in the plasma of PIH pregnant women was significantly increased (Santillan et al., 2014; Yeung et al., 2014), indicating that the release of vasopressin may be a predictor of PIH. Vasopressin is an effective systemic vasoconstrictor. A large amount of vasopressin in the blood circulation can cause significant vasoconstriction. Compared with other vasoconstrictors, vasopressin induces a relatively mild vasoconstriction response in placental blood vessels. Wareing, et al. found that compared with the normal placenta, the placenta of PIH has a significantly lower sensitivity to vasopressin contraction (Wareing and Baker, 2004), while the study of Ong, et al. showed that the contractile response caused by vasopressin is no different in PIH and normal pregnancy (Ong et al., 2002). Our recent study on 84 placentas have found that compared with normal placenta, the blood vessels of the PIH placenta show a lower sensitivity to vasopressin (Gao et al., 2019).

In reviewing the research about GDM, some studies indicated that oxytocin was involved in the deterioration of glucose tolerance in GDM (Stock et al., 1993; Kontoangelos et al., 2013; Gu et al., 2021). Gu, et al. reported that the blood oxytocin levels were lower in patients with GDM than in healthy pregnant women and were associated with impaired pancreatic β-cell function (Gu et al., 2021); and administration of oxytocin increased insulin secretion in gestating mice (Gu et al., 2021). These findings provided strong evidence that oxytocin is needed for maintaining β-cell function during pregnancy, and the lack of oxytocin could be associated with the risk of GDM. Vasopressin is suggested to be involved in gestational diabetes insipidus (Aleksandrov et al., 2010; Christ-Crain et al., 2021). Christ-Crain, et al. reported that gestational diabetes insipidus resulting from an increase in placental vasopressinase and finally primary polydipsia, which involves excessive intake of large amounts of water despite normal vasopressin secretion (Christ-Crain et al., 2021). Currently, there is still a lack of research on whether the changes in oxytocin and vasopressin in GDM patients cause vasoconstriction and diastolic changes.

## Summary and outlook

By reviewing previous studies on the placenta and its blood vessels in PIH and GDM, the following conclusions can be drawn: 1) Compared with non-placental blood vessels, placental blood vessels show their own unique contraction characteristics. 2) Although controversy still exists, the ability of placenta to secrete vasoactive substances and the vasoconstriction sensitivity of placental blood vessels were changed under PIH or GDM pathological conditions. A major reason for the controversial conclusions regarding the pathological changes of PIH and GDM placentas may be the small sample size. Due to the huge differences in human genetics, environment, lifestyle, etc., the number of experimental samples in many studies was too small (usually only 10), which is not enough to draw reliable conclusions on the physiological and pathological characteristics of the placenta. The other main reason for the controversial conclusions is gestational week. Compared with normal pregnancy, the gestational week of PIH and GDM is generally smaller. This confounder may be an important reason for the conflicting results of previous studies. Furthermore, the influence of maternal age and other potential co-morbidities such as obesity cannot be ignored. Although most studies attempted to exclude these confounders as much as possible, the lack of samples made some conflicting results unavoidable. The impacts of these difference are unknowable. In addition, the impact of complications such as multiple births, abnormal amniotic fluid volume and abnormal umbilical cord has not been explained by related studies. In short, future research is urgently needed to address these controversies.

Currently, agents or drugs associated with these endogenous vasoconstrictors were reported to have the therapeutic potential to PIH or GDM. For example, ET-1 receptor antagonists may have potential for the treatment of PIH (Bakrania et al., 2017). AT1R agonistic autoantibodies as a potential predictive factor and therapeutic target in PIH (Qu and Khalil, 2020). Vasopressin is suggested to be involved in gestational diabetes insipidus (Christ-Crain et al., 2021). Treatment with desmopressin is very effective on transient diabetes insipidus of pregnancy and also on preexisting or acquired central diabetes insipidus (Christ-Crain et al., 2021). In light of this, precision medicine aiming at certain genes or molecules targeting certain symptoms or diseases requires the precise pathophysiology to be known. Current research on the placental vascular function changes of PIH and GDM is still very limited, and the physiological characteristics of human placental blood vessels and the pathological changes in the state of PIH and GDM are worth further investigation.
